# Margin matters: analyzing the impact of circumferential margin involvement on survival and recurrence after incomplete total mesorectal excision for rectal cancer

**DOI:** 10.1007/s10151-024-03098-9

**Published:** 2025-01-23

**Authors:** A. Alipouriani, F. Almadi, D. R. Rosen, D. Liska, A. E. Kanters, K. Ban, E. Gorgun, S. R. Steele

**Affiliations:** 1 Department of Colorectal Surgery, Digestive Disease Institute, Cleveland Clinic, Cleveland, OH USA; 2 Department of Colon and Rectal Surgery, Cleveland Clinic Main Campus Submarket, Cleveland Clinic Foundation, 9500 Euclid Avenue, Cleveland, OH 44195 USA

**Keywords:** Incomplete TME, Rectal adenocarcinoma, Mesorectal excision, Margin status, Neoadjuvant therapy

## Abstract

**Background:**

Incomplete mesorectal excision during rectal cancer surgery often leads to positive circumferential margins, with uncertain prognostic impacts. This study examines whether negative margins can mitigate the poorer prognosis typically associated with incomplete total mesorectal excision (TME) in rectal cancer surgery, thus potentially challenging the prevailing emphasis on complete mesorectal excision.

**Patients and methods:**

A retrospective analysis was conducted on patients who underwent proctectomy for rectal adenocarcinoma with incomplete TME at a single center from 2010 to 2022. Patients were stratified by margin status as determined by pathologic analysis into three groups: involved, not involved with closest margin distance ≤ 2 mm, and not involved with closest margin distance > 2 mm. Outcomes included recurrence and survival. Effects of neoadjuvant therapy protocols on margin status were also assessed.

**Results:**

From 2010 to 2022, 7941 patients underwent proctectomy for rectal cancer, with 236 (3%) having incomplete TME. The median age of these patients was 64 years, and 63% were male. Overall, margin involvement was observed in 54 (23%) patients. The median tumor size was 3.05 cm (interquartile range (IQR): 2–6) for the whole group. Involved margins (23.2%) had reduced overall survival (60.5 months versus 87.3 months, *p* < 0.001), increased local recurrence (20.4% versus 9.4%, *p* = 0.024), and lower disease-free survival (45.2 versus 58.9 months, *p* = 0.006) versus uninvolved margins. Margin involvement was prognostic for decreased survival even after adjusting for confounders (*p* < 0.05). Among uninvolved margins, distance (> 2 mm versus ≤ 2 mm) did not affect outcomes. Total neoadjuvant therapy (versus standard chemoradiation) was associated with lower involved margins (*p* = 0.007).

**Conclusions:**

Positive margins retain negative prognostic impact with incomplete TME. Optimization of surgical resection remains vital. Total neoadjuvant therapy was associated with a lower rate of margin involvement.

## Introduction

Rectal cancer accounts for approximately 30% of colorectal cancers, with over 45,000 new cases and 15,000 deaths estimated in the USA in 2023 [1, 2]. Total mesorectal excision (TME) has revolutionized rectal cancer surgery, as the completeness of resection is critical for superior outcomes [3, 4]. During TME, the mesorectum and regional lymph nodes are removed along the mesorectal fascia, but anatomical constraints or advanced tumors can result in incomplete TME, characterized by breaches or fragmentation [5, 6]. This occurs in 5–30% of resections and significantly increases the risk of positive circumferential margins (CRM) and poorer outcomes [7–9]. While positive margins are known to worsen prognosis, their specific implications in incomplete TME remain unclear, highlighting the need for further investigation [10]. Negative margins may mitigate adverse outcomes when complete TME is not feasible owing to advanced tumors or anatomical limitations [11, 12]. However, achieving margin negativity in incomplete TME cases can be challenging, and while negative margins may reduce risks, they are not a substitute for complete TME. This study examines whether negative margins can offset poor outcomes in incomplete TME, contributing to the evolving understanding of the balance between margin status and TME integrity.

Recent years have seen a shift in preoperative management with the introduction of neoadjuvant chemoradiotherapy (nCRT) and total neoadjuvant therapy (TNT) [13, 14]. These treatments aim to reduce tumor size and improve resectability, potentially leading to better margin statuses. Standard chemoradiation involves a shorter course of radiation combined with chemotherapy before surgery to control local disease and enhance resection success [15]. TNT, a more intensive regimen combining full chemotherapy and chemoradiation, shows promise in improving pathological response and sphincter preservation in distal tumors, though further studies are needed [16, 17]. The specific impact of these therapies on patients undergoing incomplete TME remains uncertain.

The poorer prognosis associated with incomplete mesorectal excision warrants analysis of perioperative outcomes in this high-risk population [15–18]. This study evaluates the prognostic impact of positive surgical margins in patients with incomplete TME, hypothesizing that positive margins are linked to higher recurrence and reduced survival. It also examines margin positivity rates across neoadjuvant therapy regimens and explores the influence of margin distance and neoadjuvant treatment on prognosis, aiming to optimize management strategies for this group.

## Patients and methods

### Study design

This was a retrospective cohort study performed at a quaternary referral center. Patients who underwent surgical resection for primary rectal adenocarcinoma with reported incomplete total mesorectal excision between January 2010 and December 2022 were identified. Incomplete TME determination was based on documentation of residual mesorectum by pathology evaluation of the surgical specimen in the synoptic report. The study was approved by the institutional review board (IRB no. 23–798).

### Participants

We included adult patients (≥ 18 years) with rectal adenocarcinoma who underwent proctectomy with incomplete TME, documented on final pathology. TME was defined by residual mesorectum, graded as complete, partial, or incomplete on the basis of pathology standards. This study focused on the high-risk incomplete TME group owing to its strong association with margin involvement and adverse outcomes. Partial TMEs were excluded owing to variability in interpretation and outcomes, and complete TMEs were excluded owing to their established superior results. All surgical approaches (open, laparoscopic, and robotic) were eligible, but patients with complete or near-complete TME or surgery for rectal cancer recurrence were excluded. Patients with stage 4 cancer were included to provide a comprehensive view of outcomes across all rectal cancer stages.

### Data collection

This retrospective study collected data from electronic medical records, entered into a secure database. Key variables included demographics (age, sex, race, and BMI), clinical data (American Society of Anesthesiologists (ASA) classification, tumor location, and stage), and treatment specifics (neoadjuvant therapy regimens, surgical procedures, and adjuvant therapy). Magnetic resonance imaging (MRI)-based staging detailed tumor stages (mrT0–mrT4b) and nodal involvement (mrN0–mrN2). Pathological data included tumor size, histological grade, margin status, and distance. Follow-up data encompassed recurrence, vital status, and last contact date. Additional variables included comorbidities, tumor height, radiologic staging, neoadjuvant treatment details (chemoradiation or TNT), timing of surgery, procedural data (operative time, intraoperative events, and hospital stay length), and postoperative complications classified by the Clavien–Dindo system.

### Exposure variables

The primary exposure variable in this study was surgical circumferential margin status which was determined by pathological analysis after surgery. It was stratified into three distinct groups: (1) involved margin, defined as tumor cells present microscopically at the inked edge, (2) uninvolved margins less than or equal to 2 mm, and (3) uninvolved margins greater than 2 mm. We chose a 2 mm cutoff to categorize margin groups on the basis of existing data showing distances under 2 mm have highest risk of recurrence, without evidence of further discrimination between < 1 mm versus 1–2 mm groups [19–21].

Patients were categorized on the basis of the closest microscopic margin distance reported by the pathologist. Pathology reports included mesorectal completeness grade, TNM stage, and circumferential margin status. A “complete mesorectum” required an intact mesorectal envelope with a smooth fascia and no defects deeper than 5 mm. An “incomplete mesorectum” indicated defects exposing muscularis propria and/or irregular circumference.

### Outcome variables

The primary outcomes were local recurrence and overall survival. Local recurrence was defined as tumor regrowth in the pelvic cavity, excluding peritoneal recurrence. Overall survival was measured from surgery to death from any cause. Secondary outcomes included systemic recurrences (distant metastases), total recurrence rate (local or systemic), and time to detection of local or systemic recurrence. Disease-free survival was defined as the interval from surgery to any recurrence or death.

Margin-specific outcomes included margin positivity rates across neoadjuvant therapy groups and overall survival comparison based on the three-tiered margin categories (involved, uninvolved ≤ 2 mm, and uninvolved > 2 mm). Subgroup analysis evaluated oncologic outcomes between standard neoadjuvant chemoradiotherapy (CRT) and total neoadjuvant therapy (TNT), including recurrence rates and survival. Patients with incomplete TME were assessed for adjuvant therapy on the basis of disease stage, with chemoradiotherapy administered as indicated.

### Statistical analysis

Descriptive statistics summarized population characteristics by margin groups using median (IQR) or frequency (percentage). Kaplan–Meier curves illustrated survival distributions, with log-rank tests assessing differences. Cox proportional hazards models identified variables associated with mortality, and appropriate tests ensured model assumptions were met. Chi-squared analysis compared margin positivity rates across neoadjuvant therapy groups. Statistical significance was set at *p* < 0.05. Analyses were performed using SPSS version 26 (IBM, Armonk, NY).

## Results

### Patient and tumor characteristics

Between 2010 and 2022, of the 7941 patients who underwent proctectomy, 236 (3%) were identified as having incomplete TME. Table 1 outlines baseline demographics, clinical stage, neoadjuvant therapy details, surgical approach, pathology, and short-term postoperative outcomes. The cohort was predominantly male (62%). The median age was 64 years (IQR: 54–72 years). Median body mass index was 27.1 kg/m^2^ (IQR: 23–31 kg/m^2^). Over half (58.6%) had an ASA score of 3 or greater indicating a high burden of comorbid illnesses.

**Table 1:** Patient demographics, surgical details, and pathological outcomes

Variables	*N* = 236
Males	146 (62%)
Age, in years	64 (54–72)
Body mass index, kg/m^2^	27.1 (23–31)
ASA Score, ≥ 3	139 (58.6%)
Syndrome	
Crohn’s	3 (1.2%)
UC	3 (1.2%)
FAP	1 (0.4%)
Lynch	1 (0.4%)
Smokers	110 (46%)
Past abdominal surgery	84 (35.5%)
CEA (ng/ml)	2.9 (0.4 – 171.8)
Neoadjuvant chemoradiation	142 (60%)
Total neoadjuvant treatment	34 (14.5%)
Tumor heights from AV, cm	4 (2–7)
Clinical stage	
I	33 (14%)
II	65 (27.5%)
III	110 (46.5%)
IV	23 (10%)
Staging type	
mrT	
mrT0	2
mrT1	12
mrT2	47
mrT3	120
mrT4	3
mrT4a	17
mrT4b	9
mrN	
mrN0	96
mrN1	77
mrN2	20
Surgery details	
Procedure type	
APR	127 (54%)
LAR (including TaTME)	94 (39.7%)
LAR with colonic “J” pouch	8 (3.3%)
Total proctocolectomy with IPAA*	7 (3%)
Surgery type, *n* (%)	
Open	90 (38%)
Laparoscopic	123 (52%)
Robotic	24 (10%)
Stoma creation	223 (94%)
Diverting loop ileostomy (DLI)	70 (30%)
Conversion to open surgery	13 (5.5%)
Intraoperative complications	2 (0.8%)
Urethral injury	6 (2.5%)
Operative duration, min	292 (237–364)
30-day postoperative complications	
Length of stay, days	7 (5–10)
Clavien–Dindo classification ≥ 3	26 (11%)
Readmission	33 (14%)
Reoperation	18 (7.5%)
Pathology	
Distance from closest margin, mm	5 (1–10)
Distance from distal margin, cm	3 (1.5–4.5)
Positive surgical margin	54 (23%)
Tumor size, cm	3.05 (2–6)
Differentiation	
Poor	33 (14%)
Moderate	95 (40%)
Well	33 (14%)

Figures represent the median (interquartile) or frequency (proportion)

*UC* ulcerative colitis, *FAP* familial adenomatous polyposis

Prior to surgery, 142 patients (60%) received neoadjuvant chemoradiation therapy and 33 patients (14%) were treated with total neoadjuvant therapy consisting of chemotherapy followed by chemoradiation. Regarding cancer stage among patients with incomplete TME, 33 patients (14%) had stage I disease, 65 patients (27.5%) had stage II, 110 patients (46.5%) had stage III, and 23 patients (10%) had stage IV disease. The median distance of the tumor from the anal verge was 4 cm (IQR:2–7). The median distance from distal margin in our cohort was 3 cm (IQR: 1.5–4.5 cm). The distribution of tumor stages was as follows: mrT0 (*n* = 2), mrT1 (*n* = 12), mrT2 (*n* = 47), mrT3 (*n* = 120), mrT4 (*n* = 3), mrT4a (*n* = 17), and mrT4b (*n* = 9). For nodal involvement, the data showed: mrN0 (*n* = 96), mrN1 (*n* = 77), mrN2 (*n* = 28.

### Surgical approach and perioperative details

The most common surgical procedure performed was abdominoperineal resection (APR), conducted in 128 patients (54%). The next most frequent was low anterior resection (LAR), which, including the transanal total mesorectal excision (TaTME) approach, was performed in 94 patients (39.7%). Most surgeries were minimally invasive, with laparoscopic procedures accounting for 52% (123 surgeries) and robotic surgeries for 10% (24 surgeries), while open surgeries constituted 38% (90 surgeries). Median operative duration was 292 min (IQR: 237 to 364 min). Intraoperative complications occurred in 70 patients (30%). Median postoperative length of hospital stay was 7 days (IQR: 5–10 days).

### Postoperative and pathological outcomes

Rates of 30-day readmission and reoperation were 14% and 7.5%, respectively. The most frequent postoperative complications included surgical site infections (16%) and ileus (6%). As per Clavien–Dindo severity grading, 11% experienced complications of grade 3 or higher in the 30 days after surgery. On final pathology evaluation, margin involvement was observed in 54 patients (23%). Specifically, close margin status fell into three distinct categories: involved margins were found in 54 patients, margins ≤ 2 mm were seen in 21 patients, and margins > 2 mm were present in 136 patients. Median tumor size was 3.05 cm. Histological grading identified 33 tumors (14%) as poorly differentiated.

### Impact of margin status on oncological outcomes (Table 2)

#### Local recurrence

**Fig. 1:**
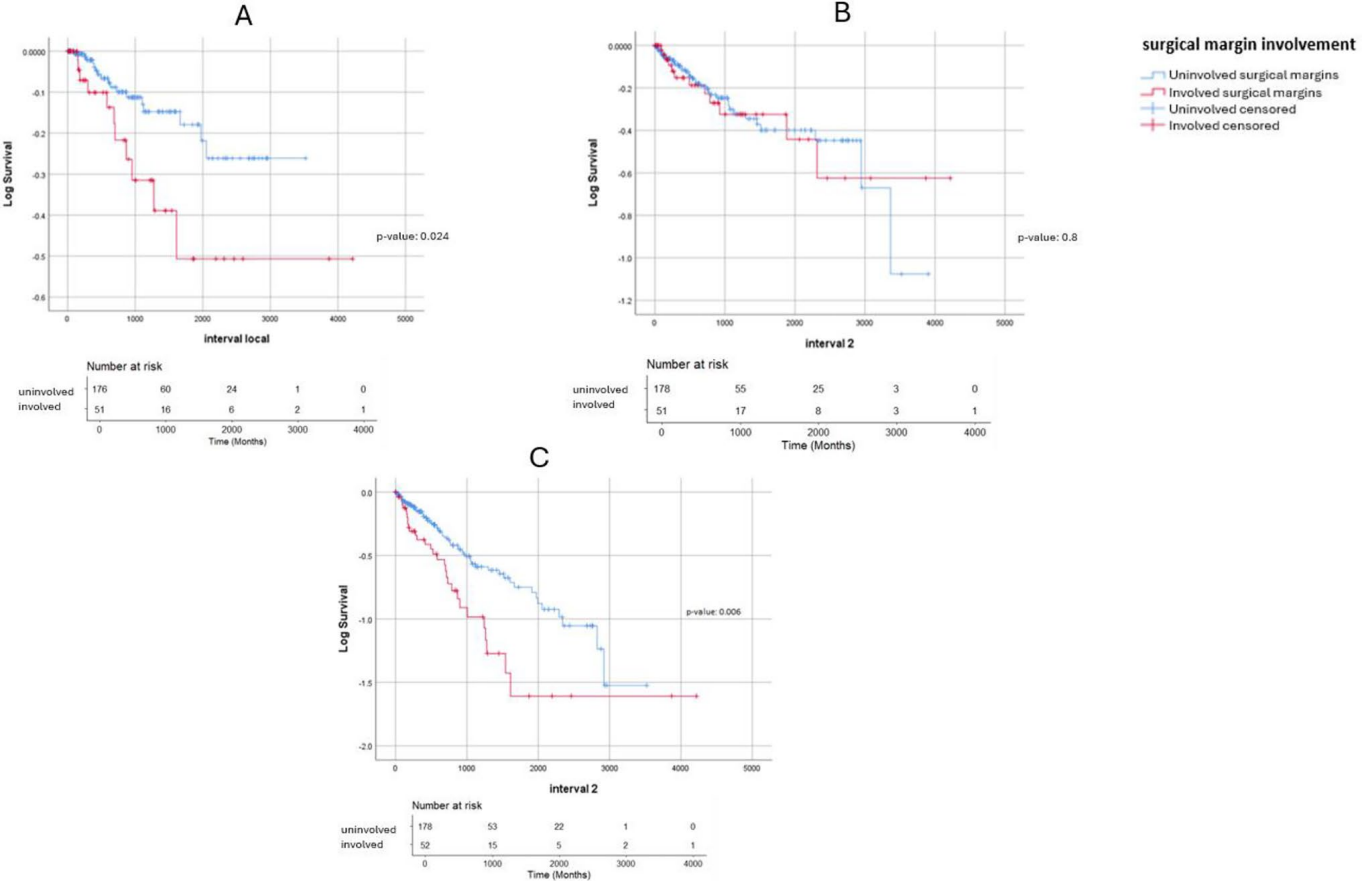
Kaplan–Meier survival curves depicting the impact of margin status on oncological outcomes. **A** Local recurrence (*p* = 0.024), **B** systemic recurrence (*p* = 0.8), **C** disease-free survival (*p* = 0.006)

Local recurrence occurred in 11 of 54 patients (20.4%) with positive margins and 17 of 180 patients (9.4%) with negative margins over the duration of follow-up. Kaplan–Meier analysis demonstrated a significantly shorter time to local recurrence for patients with positive margins compared with those with negative margins (*p* = 0.024, Fig. 1A).

**Table 2:** Impact of margin status on oncological outcomes in rectal cancer treatment

Margin status	Local recurrence (%)	Systemic recurrence (%)	Disease-free survival (DFS)	Overall survival (OS)
Positive (involved) *n* = 54	20.4	22.2	45.2 months	60.5 months
Negative (clear) *n* = 182	9.4	20.4	58.9 months	87.3 months
*p*-value	0.024	0.883	0.006	< 0.0001

#### Systemic recurrence

Systemic recurrences occurred among 12 patients (22.2%) with positive margins and 37 patients (20.4%) with negative surgical margins. No statistically significant difference in systemic recurrence rates or time to systemic recurrence was observed between the two margin groups on Kaplan–Meier analysis (*p* = 0.883, Fig. 1B).

#### Disease-free survival

Median disease-free survival was 45.2 months in the positive margin group compared with 58.9 months for patients with negative margins. Kaplan–Meier plots demonstrated reduced disease-free survival times for patients with involved margins (45.2 versus 58.9 months, *p* = 0.006, Fig. 1C) (Table 3).

**Table 3:** Cox regression analysis of overall survival

Variable	Hazard ratio (HR)	95% CI for HR	*p*-value
Age	1.021	0.999 to 1.043	0.064
Sex (male)	0.882	20.4	0.664
BMI	1.039	0.995 to 1.085	0.089
Stage	2.479	1.429 to 4.301	**0.001**
Surgical margin involvement	1.122	0.848 to 1.485	0.422

Bold value indicates *p* < 0.05

Note: In the Cox regression model, age and BMI were treated as continuous variables. The reference category for sex was male, and for surgical margin involvement, it was negative margins. Stage I was coded as the reference category

*BMI* body mass index

#### Overall survival

**Fig. 2:**
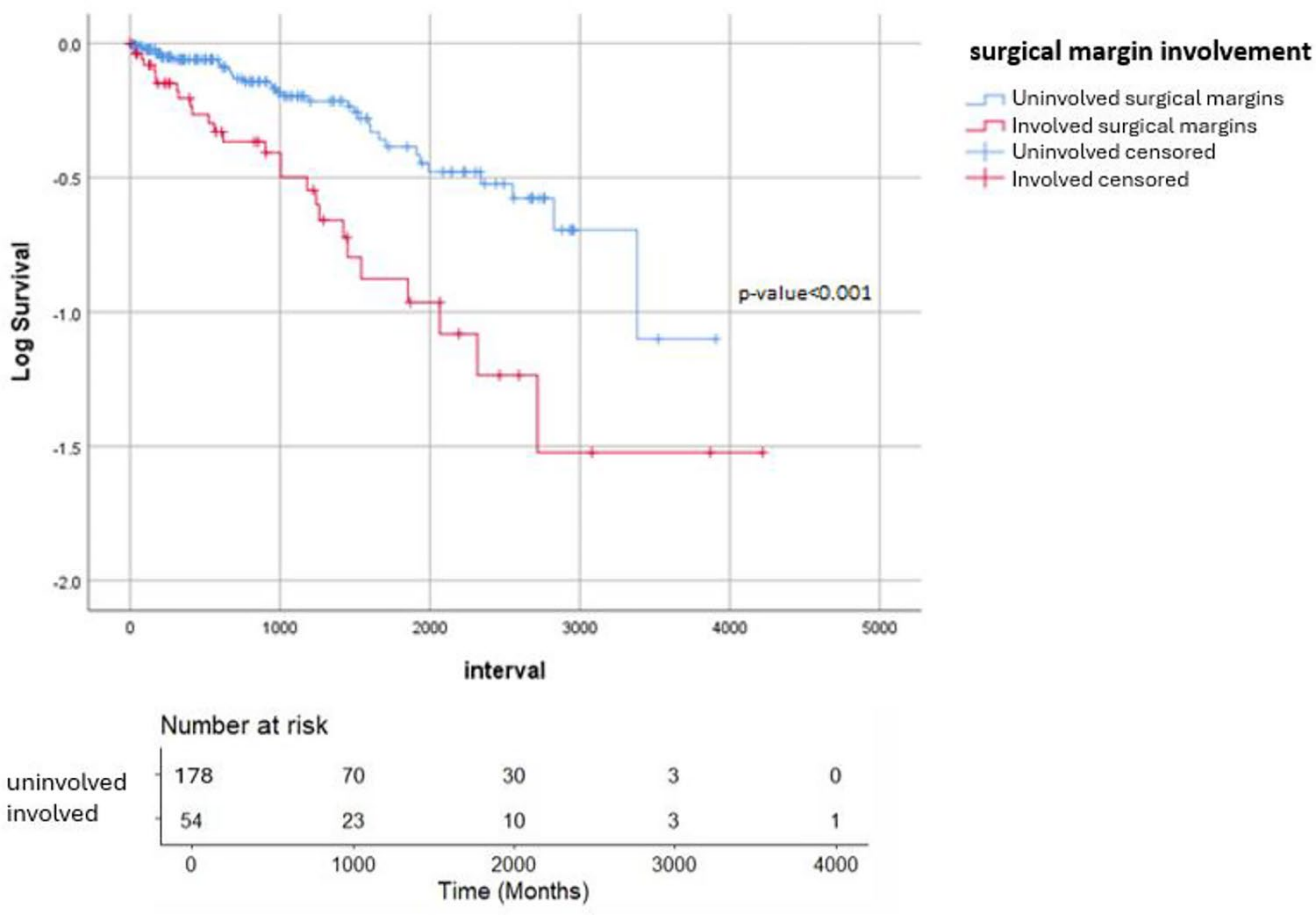
Kaplan–Meier survival curves for overall survival showing the impact of margin status on oncological outcomes

Median overall survival was 60.5 months for patients with positive surgical margins versus 87.3 months when margins were negative. Kaplan–Meier plots showed significantly reduced overall survival times associated with positive margins (log-rank *p* < 0.001). After adjusting for other variables, it was found that the stage of cancer, rather than the involvement of surgical margins, was independently associated with a higher risk of mortality (HR 2.479, 95% CI 1.4–4.3, *p* < 0.001, Fig. 2).

### Margin threshold analysis

Patients were stratified into three groups on the basis of circumferential margin status and threshold: involved margins (*n* = 54), uninvolved margins ≤ 2 mm (*n* = 21), and uninvolved margins > 2 mm (*n* = 136). Table 4 provides a detailed breakdown of patient characteristics by margin involvement status.

**Table 4:** Patient characteristics, neoadjuvant treatment, and surgical details stratified by margin involvement status

Variable	Involved (*n* = 54)	Not involved ≤ 2 (*n* = 21)	Not involved > 2 (*n* = 136)
Age, in years	63 (54–68)	65 (48–75)	66 (56–72)
Body mass index, kg/m^2^	26.08 (24.01–30.73)	28.25 (24.6–33.1)	26.57 (23.38–30.62)
Male (%)	67.9%	62%	61.3%
ASA score, ≥ 3	66.5%	70%	62%
Past abdominal surgery	35.18%	47%	36%
Tumor heights from AV, cm	3.2 (2–6.4)	3.7 (2–7.7)	4 (2.5–7)
Clinical stage			
I	2 (3.7%)	5 (23.8%)	23 (16.9%)
II	14 (25.9%)	6 (28.6%)	37 (27.2%)
III	29 (53.7%)	10 (47.6%)	60 (44.1%)
IV	8 (14.8%)	0 (0%)	15 (11%)
Unknown	1 (1.9%)	–	1 (0.8)
Neoadjuvant chemoradiation, *n* (%)	38 (70%)	14(66.6%)	76 (55.9%)
Total neoadjuvant treatment, *n* (%)	2 (3.7%)	0 (0%)	23 (16.9%)
Surgery approach (*n* = 137)			
Open	34	11	40
Laparoscopic	16	7	83
Robotic	4	3	14
Operation time, min	301 (6.25–12.75)	212 (181–268)	300 (238–364)
Length of stay, days	8 (6.25–12.75)	7 (5–10)	6 (4–10)
Readmission	8 (14.8%)	2 (9.5%)	16 (11.8%)
Reoperation	6 (11.1%)	1 (4.7%)	8 (5.9%)
Distance of invasive carcinoma from closest margin (mm)	0 (0–0)	2 (1.13–2)	8 (5–13)
Distance from distal margin (cm)	3.5 (1.5–5)	–	–

#### Survival based on three-tiered margin stratification

**Fig. 3:**
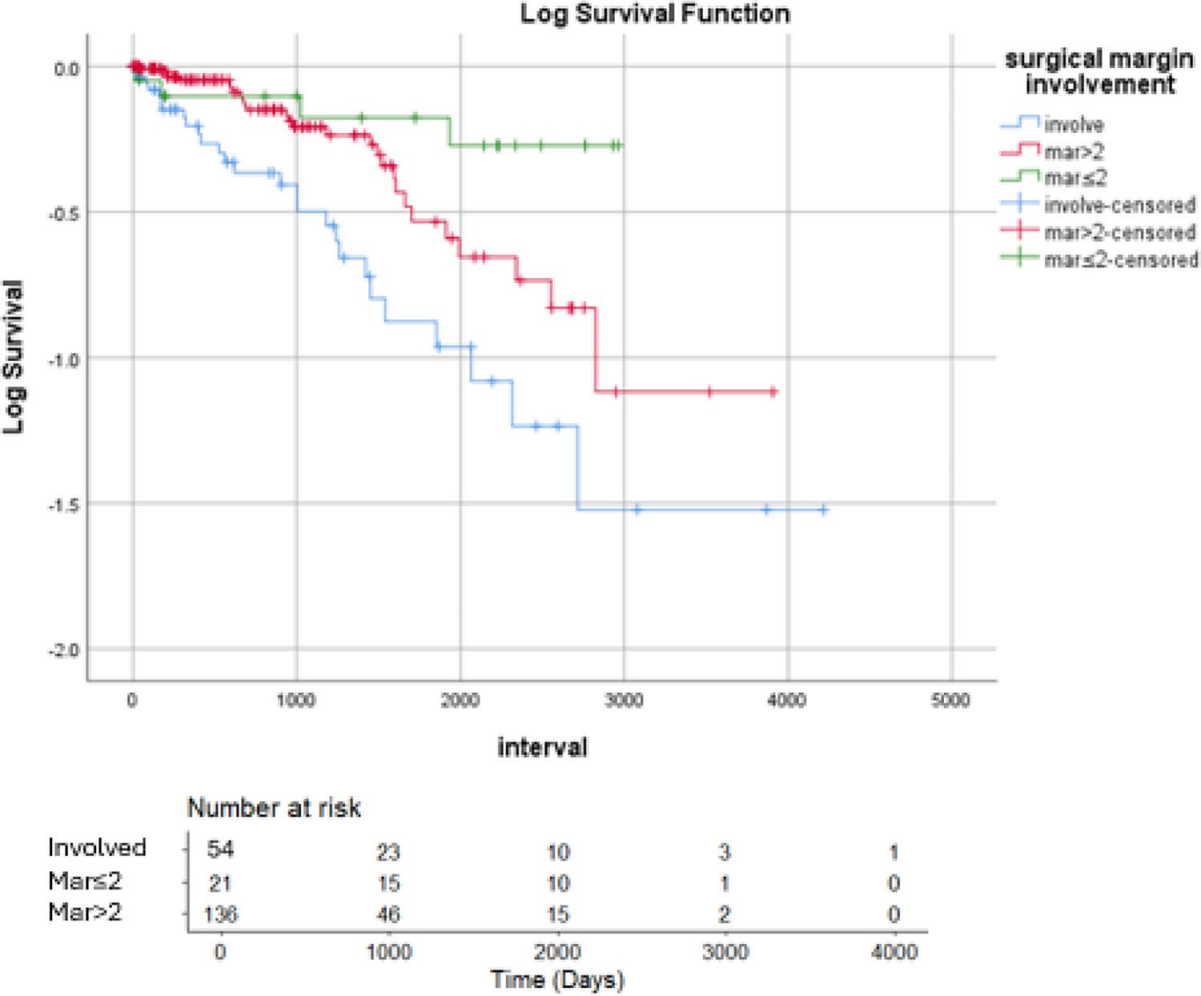
Overall survival across three-tiered margin stratification

Kaplan–Meier plots for overall survival demonstrated significantly reduced survival times for patients with involved margins compared with those with uninvolved margins > 2 mm (log-rank *p* < 0.001). When comparing the three groups, no significant difference in overall survival was seen between the uninvolved margin subgroups of ≤ 2 mm versus > 2 mm (log-rank *p* = 0.172) (Fig. 3).

On multivariate Cox regression analysis with the three-tiered margin categories, involved margin status was independently associated with an approximately 3.4-fold increased mortality risk compared with uninvolved margins > 2 mm (HR for involved versus uninvolved > 2 mm: 3.4, *p* = 0.022). No significant difference in survival was seen between uninvolved margins ≤ 2 mm versus > 2 mm (HR for ≤ 2 mm versus > 2 mm: 1.9, *p* = 0.197) after adjustment.

### Focused analysis of uninvolved margin groups

Among patients with pathologically uninvolved margins (*n* = 157), further analysis was performed comparing survival outcomes between the ≤ 2 mm (*n* = 21) and > 2 mm (*n* = 136) margin groups. However, Kaplan–Meier plots showed no significant difference in overall survival between these uninvolved margin groups (log-rank *p* = 0.172).

On multivariate Cox regression restricted to patients with uninvolved margins, close margins ≤ 2 mm were not significantly associated with mortality risk compared with margins > 2 mm (HR for ≤ 2 mm versus > 2 mm: 2.070, *p* = 0.172) after adjusting for other variables.

### Effect of neoadjuvant therapy regimens on margin

Margin involvement was assessed among patients receiving no neoadjuvant therapy (*n* = 55), standard neoadjuvant chemoradiation (neo, *n* = 130), or total neoadjuvant therapy (TNT, *n* = 25). There were no significant differences in age (*p* = 0.5), BMI (*p* = 0.8), sex (*p* = 0.2), tumor heights (*p* = 0.9), ASA scores (*p* = 0.8), past abdominal surgery (*p* = 0.4), and stage ≥ III (*p* = 0.9) between the groups (Table 5).

**Table 5:** Baseline characteristics of patients by treatment group

Characteristic	Standard neoadjuvant chemoradiation (Neo) (*n* = 128)	Total neoadjuvant therapy (TNT) (*n* = 25)	*p*-value
Age (mean ± SD)	61.58 ± 11.6	59.84 ± 11.9	0.5
BMI (mean ± SD)	27.54 ± 6.02	26.89 ± 4.98	0.5
ASA score			0.8
ASA I	7 (5.5%)	1 (4%)	
ASA II	45 (35.2%)	10 (40%)	
ASA III	70 (54.7%)	14 (56%)	
ASA IV	5 (3.9%)	0 (0.0%)	
Sex, male	83 (64.8%)	13 (52%)	0.2
Clinical stage			0.9
Stage 1	9 (7%)	2 (8%)	
Stage 2	33 (25.8%)	8 (32%)	
Stage 3	69 (53.9%)	12 (48%)	
Stage 4	16 (12.5%)	3 (12%)	

Overall, margin involvement occurred in 14 of 55 patients (26%) receiving no neoadjuvant treatment compared with 39 of 130 patients (30%) after standard neoadjuvant chemoradiation. Only 2 of the 25 patients (8%) receiving TNT had involved margins (*p* = 0.033). On focused analysis of the neoadjuvant groups, the rate of margin positivity was significantly lower after TNT (8%) versus standard neoadjuvant chemoradiation (30%, *p* = 0.007) (Table 6).

**Table 6:** Incidence of surgical margin involvement by neoadjuvant therapy type

Neoadjuvant therapy type	Number of patients	Margin involvement (*N*, %)
None	55	12 (25.5%)
Standard neoadjuvant chemoradiation (neo)	130	39 (30%)
Total neoadjuvant therapy (TNT)	25	2 (8.0%)
Total	210	55

Note: Chi-squared test indicates a significant difference in margin involvement across the three therapy groups (Pearson chi-squared *χ*^2^(2) = 10.019,* p* = 0.007; Likelihood ratio = 0.001*χ*^2^(2) = 13.121,* p* = 0.001)

Regarding oncological outcomes, local recurrence was observed in 20 patients (15.6%) in the Neo group compared with just 1 patient (4.0%) in the TNT group, although the difference in time to recurrence was not statistically significant (log-rank *p* = 0.158). Systemic recurrences occurred in 28 patients (21.7%) treated with Neo and 9 patients (36.0%) in the TNT group (log-rank *p* = 0.08). Total recurrence rates were similar between the two groups, with 34.1% in the Neo group and 36.0% in the TNT group (log-rank *p* = 0.66). Median disease-free survival was approximately 2.9 years for both groups (log-rank *p* = 0.71), and overall survival was 7.4 years for Neo patients compared with 4.4 years for TNT patients, with no significant difference (log-rank *p* = 0.32) (Table 7).

**Table 7:** Subset analysis of oncologic outcomes by neoadjuvant regimen

Outcome measure	Standard neoadjuvant chemoradiation (neo)	Total neoadjuvant therapy (TNT)	*p*-value
Local recurrence rate (*N*, %)	20/128 (15.6%)	1/25 (4%)	0.158
Systemic recurrence rate (*N*, %)	28/129 (21.7%)	9/25 (36%)	0.079
Total recurrence rate (*N*, %)	44/129 (34.1%)	9/25 (36%)	0.663
Disease-free survival (median days)	1064	1051	0.719
Overall survival (median days)	2716	1597	0.322

## Discussion

In this retrospective analysis, we demonstrated the negative prognostic effect of positive surgical margins in determining oncologic outcomes for patients with rectal cancer with incomplete TME. Patients with involved circumferential margins saw significantly higher rates of local and systemic recurrence alongside reduced overall and disease-free survival compared with those with incomplete TME and uninvolved resection margins. These adverse outcomes associated with margin positivity retained significance even after adjusting for other clinical and demographic characteristics.

The 3% rate of incomplete TME in our overall rectal cancer cohort compare favorably with the wide range of 5–30% reported in literature [6–8, 22]. Our focus on incomplete TME cases provides specific data on this high-risk subset. The 23.2% rate of positive margins in our cohort was higher than expected, likely influenced by preoperative T staging, neoadjuvant treatment selection, MRI accuracy, tumor response, and downstaging. High BMI and larger tumors may also present technical challenges during surgery [22–24]. These challenges reflect the difficulty of achieving negative margins when the mesorectal envelope is disrupted, as well as factors predisposing to incomplete TME. The margin positivity rate rose to 26% in patients without neoadjuvant therapy, though this varies across studies. For instance, a 2015 study identified positive CRM in 17.2% of 16,619 US patients undergoing resection for stage I–III rectal cancer [7]. Our results suggest negative surgical margins may offset poor outcomes associated with incomplete TME, indicating that achieving negative margins could sometimes be as critical as complete TME. Complete TME may not be feasible in cases of unfavorable anatomy, such as low rectal tumors or disrupted mesorectal envelopes. These findings highlight the need for further studies to explore surgical strategies adjusted for margin status [25].

The 60% rate of neoadjuvant chemoradiotherapy aligns with standard guidelines [26]. However, only 14.5% received TNT with either induction chemotherapy followed by chemoradiation or chemoradiation followed by consolidation chemotherapy before surgery, which has recently gained adoption for improving response. The significantly lower 8% rate of positive margins after TNT compared with 30% with chemoradiation mirrors data showing improved tumor downstaging and resectability with TNT [27]. Our findings suggest neoadjuvant protocol impacts quality of resection.

In our study, TNT was associated with a higher rate of systemic recurrences than expected. This may be owing to factors such as biological variability, tumor resistance to chemotherapy, or the presence of micrometastases that were not fully eradicated. Resistance mechanisms, including altered drug metabolism, enhanced DNA repair, or chemotherapy-resistant cancer stem cells, could contribute to this outcome. While existing literature generally shows TNT improves local and systemic control, our findings suggest the need for further investigation into factors that may influence treatment efficacy in specific patient populations [28–30].

The high APR rate (54%) in this cohort likely reflects the selection of patients with incomplete TME, who often have lower and more advanced tumors. However, most surgeries were minimally invasive, with 52% laparoscopic (123 surgeries), 10% robotic (24 surgeries), and only 38% open (90 surgeries). This highlights the rapid adoption of minimally invasive techniques and sphincter-preserving resections, which gained broader acceptance in the late 2000s [31, 32] The evolution of surgical standards from 2010 to 2022 is reflected in the increased use of minimally invasive surgery (MIS) approaches. Similarly, the overall 14.5% TNT rate obscures its growing adoption in recent years, following prospective trials in 2016–2017 [33]. This multi-year span captures the evolution of rectal cancer care. Subset analysis revealed comparable oncologic outcomes between TNT and neoadjuvant chemoradiation groups, though survival differences may require longer follow-up to become apparent.

Our 30-day readmission (14%) and reoperation rates (7.5%) are consistent with existing data on complications after proctectomy, which range from 15% to 25% in most large series [34]. This confirms the technically high-risk nature of these surgeries even without the added challenges of an incomplete TME dissection.

The 20.4% local recurrence rate for positive margins was nearly double the 9.4% rate for negative margins, with recurrence occurring significantly faster in the positive group. This supports evidence linking positive margins to higher local recurrence rates after proctectomy, typically ranging from 15% to 30% [7, 35]. Disease-free and overall survival were also significantly worse for patients with positive margins, indicating a broader impact on prognosis. Several studies emphasize the importance of resection margin status in rectal cancer surgery. A 2012 meta-analysis showed that patients with R0 resection had significantly better survival than those with R1 or R2 resections. Similarly, a 2017 systematic review found both survival and disease-free survival rates were worse in patients with positive margins after abdominoperineal excision and pelvic exenteration. These findings highlight the necessity of achieving negative margins to optimize outcomes, even when residual mesorectum is present [36, 37].

Dichotomizing margin status as positive or negative showed significant discrimination for nearly all oncologic endpoints. We also evaluated outcomes based on a 2 mm threshold, chosen because widths under 2 mm carry the highest risk, but further stratification (< 1 mm versus 1–2 mm) did not show additional prognostic value. In our study, thresholds of ≤ 2 mm versus > 2 mm provided no added discrimination beyond the positive versus negative classification, supporting the clinical utility of a binary approach. A study demonstrated that closer CRMs after preoperative chemoradiotherapy were associated with higher local recurrence and lower disease-free survival. It categorized CRM into > 2 mm, 1.1–2.0 mm, 0.1–1.0 mm, and 0 mm groups, finding worse prognosis for the closest margin (0 mm). However, survival rates were similar for margins > 2 mm and 1.1–2.0 mm [20]. This suggests very close margins significantly worsen outcomes, while slight differences around 2 mm may not.

Our study highlights the importance of meticulous surgical resection in rectal cancer, particularly addressing challenges in incomplete TME. Including patients with stage 4 cancer broadens understanding of the role of surgical margins across disease severities, aiding strategies to improve outcomes. Optimizing surgical techniques and incorporating TNT could reduce incomplete TME incidence and improve results. Limitations include the retrospective, single-center design and smaller TNT group size, potentially affecting generalizability and statistical power. While our findings emphasize the prognostic significance of CRM in incomplete TME, comparisons with complete TME outcomes, which generally show better recurrence and survival rates, were not performed. Larger studies are needed to validate these findings, compare outcomes between incomplete and complete TMEs, and identify risk factors for positive margins to enhance surgical planning.

In conclusion, our study reinforces the importance of achieving both complete TME and negative surgical margins to optimize oncological outcomes in rectal cancer surgery. In cases of incomplete TME, the presence of involved margins significantly worsens prognosis, particularly with respect to local recurrence. This reinforces the need for a multimodal approach to management, incorporating advanced preoperative therapies to optimize surgical outcomes and ultimately improve patient survival.

## Data Availability

No datasets were generated or analyzed during the current study.
